# Prevalence and Developmental Profiles of Autism Spectrum Disorders in Children With Global Developmental Delay

**DOI:** 10.3389/fpsyt.2021.794238

**Published:** 2022-01-18

**Authors:** Ling Shan, Jun-Yan Feng, Tian-Tian Wang, Zhi-Da Xu, Fei-Yong Jia

**Affiliations:** ^1^Department of Developmental and Behavioral Pediatrics, The First Hospital of Jilin University, Jilin University, Changchun, China; ^2^Department of Psychiatry, GGz Centraal, Amersfoort, Netherlands

**Keywords:** global developmental delay, Autism Spectrum Disorder, developmental profiles, Gesell, comorbidity

## Abstract

**Background:**

Previous studies have mostly explored the comorbidities of Global developmental delay (GDD) in children with Autism Spectrum Disorders (ASD) from the perspective of ASD. The study focus on the perspective of GDD to investigate the prevalence and developmental profiles of ASD in GDD and to explore the correlation between the developmental level and symptoms of autism.

**Methods:**

Clinical data of 521 children with GDD aged from 24 to 60 months were retrospectively analyzed. Analyses were performed first for the whole sample and then subdivided into two subgroups (GDD^+^ASD^−^, GDD^+^ASD^+^) according to whether had ASD. Symptoms of autism were evaluated by the Autism Behavior Checklist and the Childhood Autism Rating Scale. The Chinese version of the Gesell Developmental Schedules was used to evaluate the level of children's mental development.

**Result:**

The prevalence of ASD in children with GDD was 62.3%. The total average developmental quotient (DQ) of GDD was mildly deficient and was negatively correlated with symptoms of autism (*p* < 0.05); language ability was severe and extremely severe deficient (*P* < 0.05). GDD^+^ASD^−^ group and GDD^+^ASD^+^ group have some common points as well as differences in the developmental features. The language delay of children in both subgroups was the most obviously defected, thereafter followed by the item of personal social activity. In the GDD^+^ASD^+^ group, the DQ of gross motor skills>fine motor skills>adaptability (*p* < 0.05). There were no significant differences among the DQ of gross motor skills, fine motor skills and adaptability in GDD^+^ASD^−^ group (*p* > 0.05). The GDD^+^ASD^−^group had better adaptability, fine motor skills, language ability, personal social activity than that of the GDD^+^ASD^+^ group, but the gross motor skills in GDD^+^ASD^−^ group were worse (*p* < 0.05).

**Conclusion:**

GDD children have a high proportion of comorbid ASD, and GDD children with poorer developmental levels are more likely to have ASD symptoms. Development profiles in both GDD^+^ASD^−^ children and GDD^+^ASD^+^ children have common features but there are also differences. GDD^+^ASD^+^ group is worse than GDD^+^ASD^−^ group in terms of the overall development level.

## Introduction

Global developmental delay (GDD) is defined as a developmental disability of children under 5 years (60 months) of age, which refers to significant delays in two or more developmental domains, including gross or fine motor, speech/language, cognitive, social/personal, and activities of daily living (1). After growing up, many patients with GDD would demonstrate intellectual disability (ID) (2). It is reported in the literature that the prevalence of ID among the population receiving special education in the United States is gradually decreasing, from 8.3‰ in 2000 to 5.7‰ in 2010 (3). Autism spectrum disorder (ASD) is considered to be a neurodevelopmental disorder that can lead to severe social communication deficits, repetitive behaviors, and limited interests (4). In the past few decades, the prevalence of diagnosed ASD has increased dramatically, from 1/5000 in 1975 to 1/44 in 2021 (5), but the reason for this phenomenon remains unclear.

Both GDD/ID and ASD are neurodevelopmental disorders. These diseases overlap in their clinical manifestations and etiology. Comorbidities occur also quite often (6, 7). Previous studies have mostly explored the comorbidities of GDD/ID in children with ASD from the perspective of ASD (5, 8). Our previous studies showed that 68.3% of children with ASD under 5 years of age have combined GDD (9). Studies by the Centers for Disease Control and Prevention (CDC) in the United States showed that ~1-third of 8-year-old children with ASD have combined ID (5, 10). Few studies have explored the comorbidities and developmental characteristics of ASD in children with GDD/ID from the perspective of GDD/ID. Some scholars believe that the increase in the prevalence of ASD in recent years may be related to the lack of understanding of ASD in the past and misdiagnosis of ASD as GDD/ID; or in case of GDD/ID combined with ASD, only GDD/ID is diagnosed, but ASD is not diagnosed (3). The treatment of ASD and GDD/ID are not all the same, current evidence supports that ASD should be primarily targeted at social communication skills complemented by management of abnormal behavior, but children with GDD may obtain greater benefit from structured daily routines, cognitive behavioral therapy and so on (11, 12). Early diagnosis and targeted treatment are of great significance to improve the prognosis (13–15). A small amount of studies have investigated ASD in the ID population, and found that the comorbidity rate of ASD in the ID population was 4.2–32.9%, and ID children with comorbid ASD had more severe intellectual disability than children with ID alone (16–18). There is no research yet to explore the developmental characteristics of Chinese children with GDD and their comorbidities with ASD. Therefore, this study examined the developmental level and autism symptoms of children with GDD admitted to our hospital from January 2018 to December 2019. By investigating the prevalence and developmental profiles of ASD in children with GDD, it aims to provide a basis for precise diagnosis and personalized treatment.

## Methods

### Participants

Participants were recruited by the “Early Recognition and Intervention Program for neurodevelopmental disorders in children” project as part of the National Key Research and Development Project of China. The studies involving human participants were reviewed and approved by the Ethics Committee of the First Hospital of Jilin University. Written informed consent to participate in this study was provided by the participants' legal guardian. A total of 521 children with GDD aged 24–60 months who were treated at the Outpatient Department of Developmental & Behavior Pediatrics of the First Hospital of Jilin University from January 2018 to December 2019 were included in this study. GDD was diagnosed according to the criteria of Diagnostic and Statistical Manual of Mental Disorders V (DSM-5). The Chinese version of the Gesell Developmental Schedules (GDS) was used to evaluate the neurodevelopmental symptoms. All patients were newly diagnosed cases and received no treatment before. Children with physical or sensory disabilities, epilepsy, and genetic defects or inherited metabolic diseases, such as Fragile X Syndrome, Rett Syndrome, Angelman Syndrome, Prader-Willi Syndrome, tuberous sclerosis were excluded. According to whether the child had ASD, all the 521 participants were divided into two subgroups: GDD without ASD (GDD^+^ASD^−^, *n* = 196), GDD with ASD (GDD^+^ASD^+^, *n* = 325).

### Measurements

#### Neurodevelopment Assessment

The Chinese version of GDS was used to assess the neurodevelopmental outcomes. It is a classic psychometrical scale widely used in China to evaluate the development of children aged from 16 days to 6 years old. This instrument covers the assessment of the following five domains: gross motor skills, fine motor skills, adaptability, language, and personal social activity. GDS generates individual developmental quotient (DQ) scores for each domain. The total average quotient was calculated as the average of five DQs. Our analysis classified them as follows: normal (DQ ≥ 86), borderline (DQ 76 ≤ ~ ≤ 85), mild defect (DQ 55 ≤ ~ ≤ 75), moderate defect (DQ 40 ≤ ~ ≤ 54), severe and extremely severe defect (DQ ≤ 39) (19). In our research, GDD was defined as mild, moderate, severe and extremely severe developmental delay (DQ ≤ 75) in two or more domains of GDS.

#### Evaluation of Autism Symptoms

The autism symptoms in the participating children were assessed using the Autism Behavior Checklist (ABC) and the Childhood Autism Rating Scale (CARS) (20, 21). Both ABC and CARS are widely used in China for assessing the autism symptoms.

The ABC is a 57-item questionnaire to be completed by parent(s) or guardian(s). The questionnaire covering five aspects of autism symptoms: sensory, relating, body concept and object use, language, social and selfcare. Items are scored on a 4-point scale, ranging from 0 (no problem) to 3 (severe problem). The higher the score, the more serious the problem (20, 22). The standard cut-off value was 53, and a score above 53 points indicated high probability of ASD (23).The reliability of ABC scale in the Chinese population is 0.97, validity is 1 (24). In order to control for potential differences in parental literacy, the survey was conducted through interviews with developmental pediatrician rather than asking parents or caregiver to fill out the written survey in this study.

The CARS includes 15 items for assessing ASD-related behavior. Each item was completed by the developmental pediatrician by observing subjects and interviewing parent(s) or guardian(s). A 7-point scale (1, 1.5, 2, 2.5, 3, 3.5, 4) was used for each of the 15 items and the total score was calculated by adding the 15 item scores together. Typically developing children show CARS scores below 30 (25). The higher the score, the more severe the symptoms (20). The reliability of CARS scale in the Chinese population is 0.73, validity is 0.97 (26).

#### ASD Diagnostic Evaluation

To confirm the diagnosis, all participants with an ABC score of more than 53, CARS score of more than 30 or suspected ASD were evaluated by the Autism Diagnostic Observation Schedule (ADOS). ADOS assessments were administered and scored by a trained developmental pediatrician who met requirements for research reliability. The Chinese version of ADOS used in this study was revised on the basis of the second edition of ADOS (ADOS-2) (27). The ADOS-2 is a semi-structured, play-based assessment tool used to evaluate the core features of ASD. It consists of four different modules, which are selected according to the expressive language level of the children. Each module are scored according to a specific diagnostic algorithm under two domains: social affect and restricted and repetive behaviors. Each assessment takes approximately 45 min to complete. There is a cut-off score for the total score of each module, implying confirmation of the diagnosis of ASD.

### Procedure

During the first visit to the outpatient clinic, children with signs of GDD received an initial assessment by a developmental behavior pediatrician for ~20 min. This includes the use of a self-guided general information questionnaire to investigate the current health status, birth history (antenatal, birth complication, gestation and birth weight), past medical, early developmental history, family history and a history of caregivers. Subsequently, the outpatient pediatrician would arrange an assessment checklist that includes ABC, CARS and GDS for these children. If ABC or/and CARS score was abnormal or if ASD was clinically suspected, the child would undergo an ADOS test to further confirm the diagnosis. On the day of the first visit or the following day, the parent(s) or guardian(s) of the participating children completed the ABC after receiving the guidance of a developmental behavior pediatrician in the evaluation room. Meanwhile, the pediatrician completed the CARS by observing the subjects and interviewing parent(s) or guardian(s). If the child's emotional state was good, a trained and qualified developmental behavior pediatrician would also complete the GDS on the same day. A complete GDS assessment took ~1 h to complete. During the process, the assessment was stopped when the child experienced obvious emotional reaction, a new appointment could be made, but the assessment might be completed within 1 week. The ADOS assessment was usually completed within 45 min and usually completed within 1 week of the first visit.

### Statistical Methods

SPSS Statistics, version 22.0 (IBM Corp., NY, United States) was used to analyse the whole data. The normality of the data was tested by using the Kolmogorov–Smirnov test. Continuous data were given as means ± SDs or median (P25, P75), whereas categorical data were given as number and percentages.

Chi-squared tests and non-parametric Mann–Whitney *U*-test were used to compare the demographics and clinical data differences between two subgroups. Using an approach similar to that described by Jeste et al. (28), repeated-measures analysis of variance (ANOVA) was used to analyse the comparison of five domains DQ in GDS for all participants, within subgroups and between subgroups. A Bonferroni correction was used for *post hoc* analysis to reduce the possibility for Type I errors due to multiple comparisons. Non-parametric Mann–Whitney *U*-test was used to compare the total average DQ of GDS between the two subgroups. The comparisons of all participants in the five domain DQ distributions of GDS were performed by using a chi-square goodness-of-fit test. Partial correlation was used to analyze the correlation of DQ scores and symptoms of autism in children with GDD. All tests were two-sided, with *P* < 0.05 as the significance threshold.

## Results

### Demographic and ASD Characteristics in GDD

**Figure 1:**
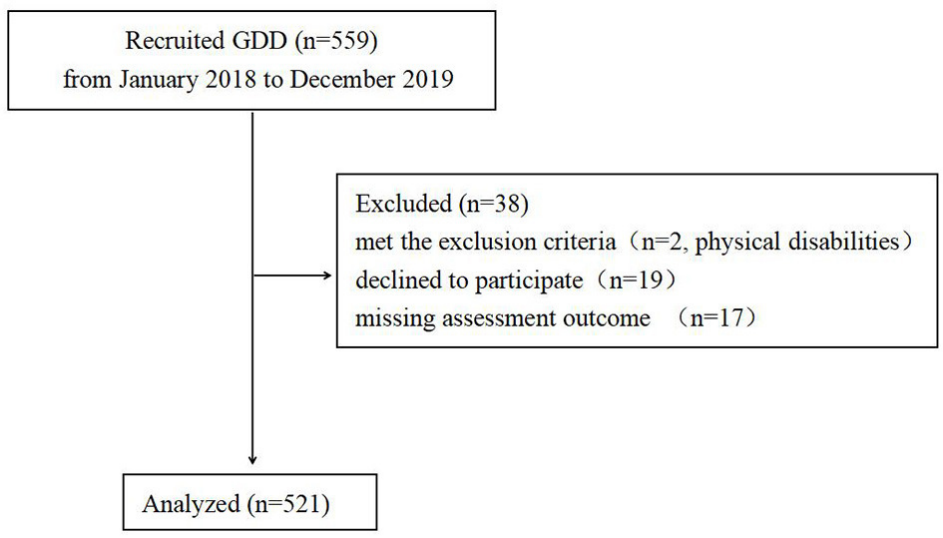
Flow chart of participants recruitment.

Patient recruitment is presented in Figure 1. A total of 521 children participated in this study. The characteristics of the study population are shown in Table 1.The average age of these children was 37.71 ± 8.58 months (range, 24–60 months). The study consisted of 427 boys and 94 girls (male-female sex ratio of 4.5:1). The proportion of ASD in GDD was 62.3% (325/521). There were 196 children in the GDD^+^ASD^−^ group (mean age = 38.27 ± 8.40 months, 162 Male/34 Female), and 325 children in the GDD^+^ASD^+^group (mean age =37.37 ± 8.68 months, 265 Male/60 Female). Both groups were matched by actual age and gender ratio (*p* > 0.05).

**Table 1:** The characteristics of the study population.

	**GDD (*n* = 521)**	**GDD^**+**^ASD^**−**^(*n* = 196)**	**GDD^**+**^ASD^**+**^(*n* = 325)**	**u/χ2**	***p***
Male/female	427/94 (4.5:1)	162/34 (4.7:1)	265/60 (4.4:1)	0,103	0.749
Age at diagnosis (months)	37.0 (31.0, 43.0)	38.0 (33.0, 44.7)	35.1 (30.0, 42.8)	−1.598	0.110
Total ABC score	48.0 (36.0, 60.0)	40.0 (27.2, 51.7)	53.0 (42.0, 64.0)	8.259	<0.001
Body concept and object use	10.0 (5.0, 14.0)	8.0 (4.0, 13.0)	10.0 (7.0, 15.0)	3.513	<0.001
Sensory	7.0 (4.0, 10.0)	5.0 (2.0, 8.0)	8.0 (5.0, 11.5)	7.152	<0.001
Selfcare	11.0 (8.0, 14.0)	9.5 (6.0, 13.0)	12.0 (9.0, 15.0)	5.456	<0.001
Language	7.0 (4.0, 10.0)	5.0 (3.0, 8.7)	7.0 (5.0, 10.0)	4.718	<0.001
Relating	12.0 (7.0, 16.0)	10.0 (4.0, 13.0)	14.0 (10, 17.0)	7.062	<0.001
Total CARS score	31.0 (27.7, 34.0)	28.0 (26.0, 31.0)	32.0 (29.5, 35.5)	9.559	<0.001

*GDD, Global developmental delay; ASD, Autism Spectrum Disorder; ABC, the Autism Behavior Checklist; CARS, the Childhood Autism Rating Scale; Chi-squared tests and non-parametric Mann–Whitney U-test were used to compare the demographics between GDD^+^ASD^−^ group and GDD^+^ASD^+^group; p < 0.05 indicated statistical significance*.

### Developmental Features of GDD

**Figure 2:**
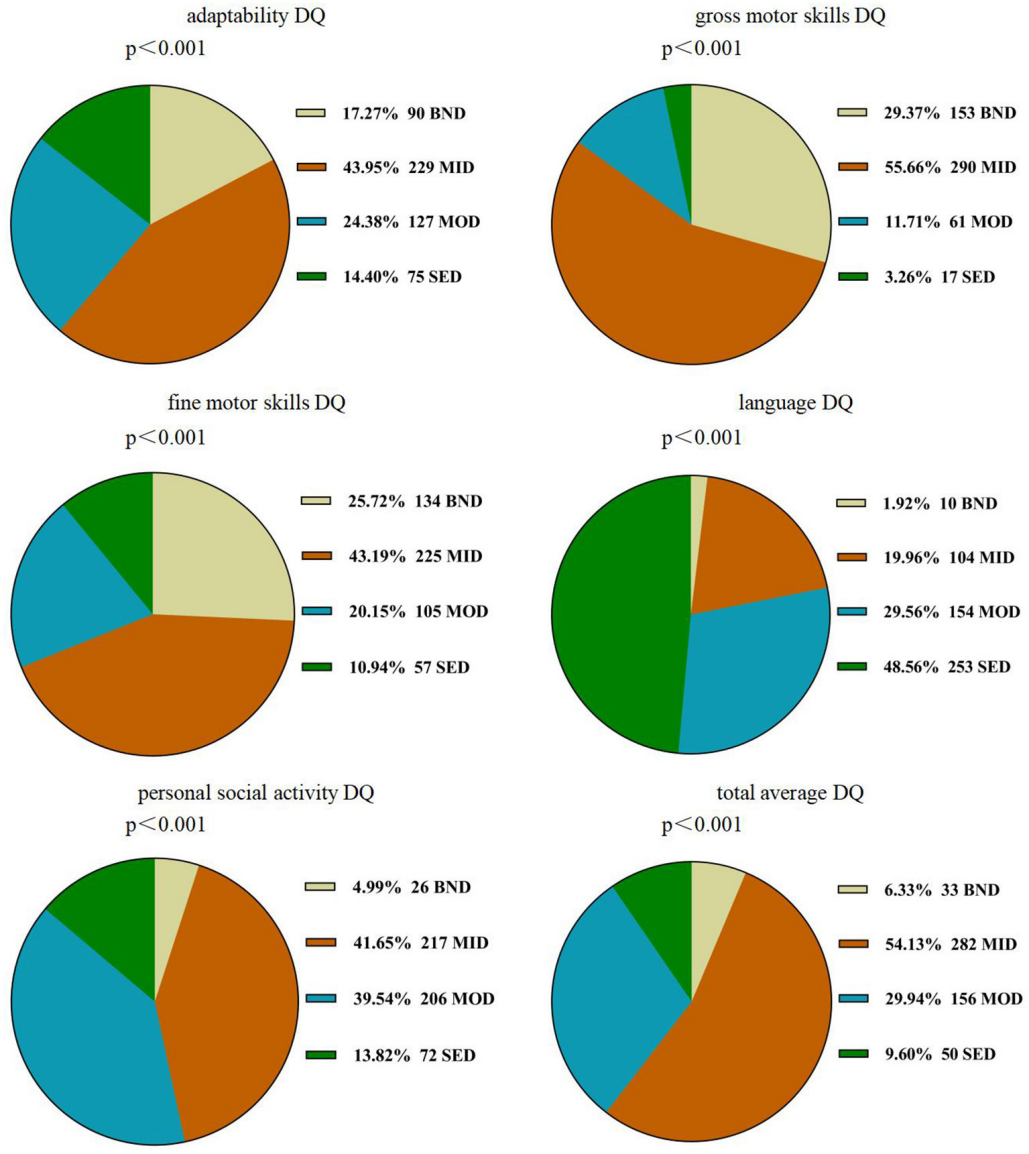
Distribution of DQ scores of GDS in children with GDD. GDD, Global developmental delay; DQ, developmental quotient; GDS, the Gesell Developmental Schedules; BND, borderline-normal defect; MID, mild defect; MOD, moderate defect; SED, severe and extremely severe defect.

A repeated-measures ANOVA showed a significant difference within the five subscale quotients of the GDS (*F* = 311.726, *P* < 0.001,η^2^ = 0.707), indicating the existence of an unbalanced development of GDD. Language delay was the most common defect (98.1%), followed by personal social skills (95.0%), adaptability (82.9%), fine motor (74.3%), gross motor (67.4%) (see also Table 2). The chi-square goodness-of-fit test showed that the language DQ was mainly dominated by the distribution of severe and extremely severe defects (χ2 = 236.321, *P* < 0.001). The DQ of the remaining four regions and the total average DQ were mainly mild defects (χ2_adaptability_ = 110.823, *p* < 0.001; χ2_grossmotor_ = 335.192, *p* < 0.001; χ2_finemotor_ = 115.123, *p* < 0.001; χ2_personalsocialskills_ = 211.322, *p* < 0.001; χ2 _totalaverageDQ_ = 303.944, *p* < 0.001) (see Figure 2).

**Table 2:** GDS scores of all the participants in different groups.

	**GDD (*****n*** **=** **521)**		**GDD**^**+**^**ASD**^**−**^**(*****n*** **=** **196)**		**GDD**^**+**^**ASD**^**+**^ **(*****n*** **=** **325)**		***p***
	**Delay^a^ *n* (%)**	**DQ (Mean ±SD)**	**Delay^a^ *n* (%)**	**DQ (Mean ±SD)**	**Delay^a^ *n* (%)**	**DQ(Mean ±SD)**	
Adaptability	432 (82.9)	59.17 ± 17.41	139 (70.9)	64.32 ± 18.82	293 (90.2)	56.06 ± 15.73	<0.001
Gross motor skills	351 (67.4)	68.32 ± 13.70	131 (66.8)	66.17 ± 14.38	220 (67.7)	69.61 ± 13.13	0.005
Fine motor skills	387 (74.3)	63.79 ± 18.06	136 (69.4)	66.46 ± 18.13	251 (77.2)	62.18 ± 17.85	0.009
Language	511 (98.1)	42.26 ± 15.56	190 (96.9)	45.42 ± 15.28	321 (98.8)	40.36 ± 15.44	<0.001
Personal social activity	495 (95.0)	53.62 ± 13.23	182 (92.9)	56.64 ± 13.50	313 (96.3)	51.80 ± 12.75	<0.001
Total average quotient	488 (93.7)	57.43 ± 12.45	180 (91.8)	59.80 ± 12.86	308 (94.8)	56.00 ± 11.99	<0.001

*GDD, Global developmental delay; ASD, Autism Spectrum Disorder; DQ, developmental quotient; GDS, the Gesell Developmental Schedules;*

a *total average quotient or a subscale quotient ≤ 75; The repeated-measures ANOVA, Bonferroni's multiple comparison test and Non-parametric Mann–Whitney U-test were used to compare the differents between the GDD^+^ASD^−^ group and GDD^+^ASD^+^group, p < 0.05 indicated statistical significance*.

### The Association Between DQ and Autistic Symptoms in Children With GDD

After controlling gender and age, partial correlation analysis showed that the total scores of the ABC and the CARS were negatively correlated with GDS adaptability DQ, fine motor DQ, language DQ, personal social skills DQ, and total average DQ. The total scores of CARS was negatively correlated with gross motor DQ, but there was no correlation between the total scores of the ABC and gross motor DQ (see Table 3).

**Table 3:** The association between developmental quotient of GDS and symptoms of autism in children with GDD.

	**Total ABC score**	**Total CARS score**
Adaptability	r_s_ = −0.176, *p* <0.001	r_s_ = −0.276, *p* <0.001
Gross motor skills	r_s_ = −0.022, *p* = 0.619	r_s_ = −0.120, *p* = 0.006
Fine motor skills	r_s_ = −0.145, *p* = 0.001	r_s_ = −0.276, *p* <0.001
Language	r_s_ = −0.151, *p* = 0.001	r_s_ = −0.366, *p* <0.001
Personal social activity	r_s_ = −0.275, *p* <0.001	r_s_ = −0.427, *p* <0.001
Total average quotient	r_s_ = −0.193, *p* <0.001	r_s_ = −0.383, *p* <0.001

*GDD, Global developmental delay; GDS, the Gesell Developmental Schedules; ABC, the Autism Behavior Checklist; CARS, the Childhood Autism Rating Scale; rs was partial correlation coefficients; When P < 0.05, the correlation parameters can take negative values indicating negative correlation and positive values for positive correlations*.

### Comparison of the Developmental Features of the Two Subgroups

**Figure 3:**
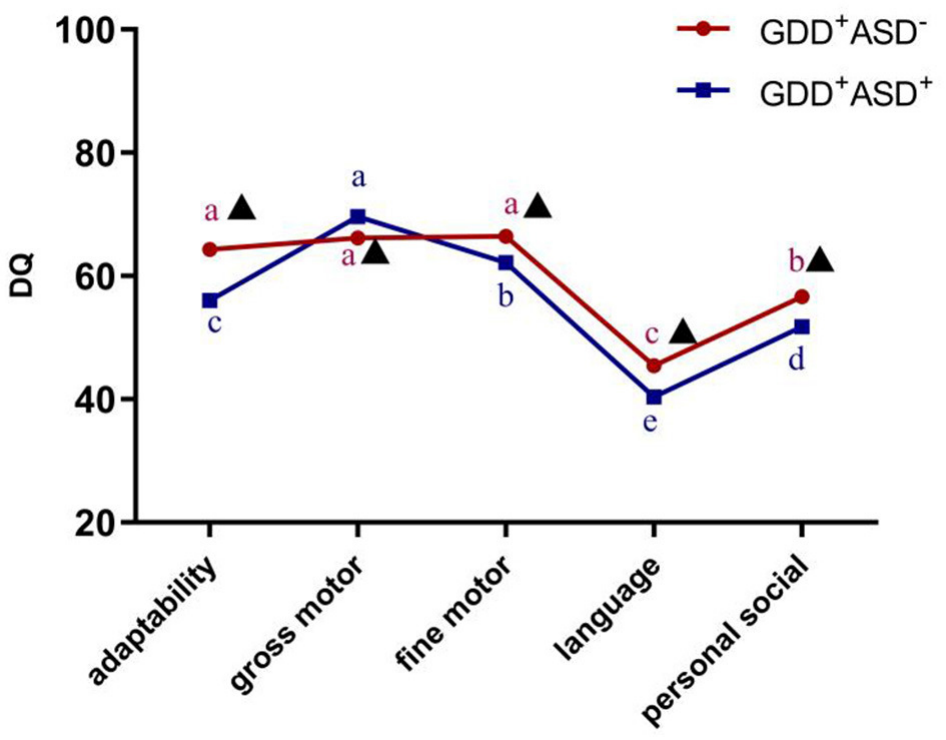
Development profile of the Gesell Developmental Schedules in GDD^+^ ASD^−^ group and GDD^+^ ASD^+^ group. GDD, Global developmental delay; ASD, Autism spectrum disorder; DQ, developmental quotient; Mean values marked with different letter (a, b, c, d or e) indicate DQ scores in the five sub-domains of Gesell Developmental Schedules, and those with the same letter exhibit no statistically significant difference while different letters represent with statistical difference (comparison within the group); ▴Indicates that there is a significant difference between GDD^+^ASD^−^ and GDD^+^ASD^+^ group (comparison between groups); *p* < 0.05 indicated statistical significance.

According to the results of the repeated-measures ANOVA, there was a significant interaction between DQ in the five sub-regions of GDS and the diagnosis (*F* = 22.305, *P* < 0.001,η^2^ = 0.147), indicating that GDD^+^ASD^−^ group and GDD^+^ASD^+^ group might have different development quotient profiles. After Bonferroni's multiple comparison test, it is found that the distribution trends of the DQ of gross motor, fine motor and adaptability were different, although the two subgroups were both dominated by language delay, followed by personal social backwardness. The GDD^+^ASD^+^ group showed a pattern of gross motor DQ > fine motor DQ > adaptability DQ (*P* < 0.001); there was no statistical difference among the DQ of gross motor, fine motor and adaptability in the GDD^+^ASD^−^group (see Figure 3).

### Description of the Developmental Features of Two Subgroups

According to the results of the repeated-measures ANOVA, Bonferroni's multiple comparison test and Non-parametric Mann–Whitney *U*-test, it is found that the GDD^+^ASD^+^ group scored lower in the DQ of adaptability, fine motor, language, personal social skills and total average DQ compared to the GDD^+^ASD^−^ group (p_adaptability_ < 0.001, p_finemotor_ = 0.009, p _language_ < 0.001, p_personalsocialskills_ < 0.001, p _totalaverageDQ_ < 0.001), and gross motor DQ was higher than GDD^+^ASD^−^ group (*p* = 0.005) (see Table 2).

## Discussion

In this study, we analyzed the developmental characteristics of 521 children with GDD and comorbid autism symptoms aged from 24 to 60 months. The development profile of GDD children with or without ASD was described and compared. The relationship between developmental level of children with GDD and the symptoms of autism was also discussed.

The results obtained are summarized as follows: 1. The comorbidity rate of ASD in children with GDD at 24–60 months was 62.3%. 2. Developmental imbalances was shown in the overall GDD children. The DQ of adaptability, gross motor, fine motor, personal social skills and total average DQ were dominated by mild defects, while language DQ was dominated by severe and extremely severe defects. 3. GDD Children with poor developmental levels were more likely to have symptoms of autism. 4. The GDD^+^ASD^−^ group and GDD^+^ASD^+^ group had some similarities in the developmental features, and there were also some differences. Language delay was the most obvious defect of both groups of children, followed by personal social activity; In the GDD^+^ASD^+^ group, the DQ of gross motor>fine motor>adaptability. There were no significant differences between gross motor DQ, fine motor DQ and adaptability DQ in the GDD^+^ASD^−^ group. 5. The GDD^+^ASD^−^ group had better adaptability, fine motor skills, language ability, personal social activity than GDD^+^ASD^+^ group, but their gross motor skills were worse.

### Prevalence of ASD in Children With GDD

Our study is the first report on the comorbidity of ASD among children with GDD in China. We found that the comorbidity rate of ASD in children with GDD aged 24–60 months was 62.3%, which is significantly higher than the 1% prevalence of ASD in the Chinese general population (29), confirming that GDD is a “high risk” sample of ASD. In addition, previous studies have also found that the proportion of GDD in children with ASD was as high as 68.3% (9), indicating that GDD and ASD have a high co-morbidity rate. Several studies have shown that the increase in the prevalence of ASD was accompanied by a decrease in the prevalence of ID (3), suggesting that the diagnosis of ASD in children with ID might be related to the increase in the prevalence of ASD. Although there is no research on the correlation between the prevalence of GDD and the prevalence of ASD, GDD is closely related to ID. The confirming diagnosis of comorbid ASD in children with GDD might also be one of the reasons for the rapid increase in the prevalence of ASD. Previous studies have found that the proportion of ASD in ID was about 4.2–32.9% (5, 16–18), which is much lower than the 62.3% ASD comorbidity rate in GDD found in this study. This phenomenon, on the one hand, may be related to the changes in the clinical phenotype of neurodevelopmental disorders with age and maturity of the central nervous system (30). On the other hand, it also indicates that the diagnosis of ASD may be insufficient. Some scholars have found that in the past, about 50% of children with ID comorbid ASD did not get a clear ASD diagnosis (31, 32). It is emphasized that clinicians need to pay more attention to and strengthen the early recognition and diagnosis of ASD comorbidities in GDD/ID. The targeted treatment should also be given to improve the prognosis.

### Developmental Features of GDD

Concerning the development profile in children with GDD, we found that the total average DQ of GDD was mainly mildly defect, which is similar to the results of previous studies (33, 34). Comparing the DQ scores on the five sub-scale of GDS, it was found that children with GDD had developmental imbalance, which was different from the previous conclusion that GDD was considered to be an overall delay in development rather than developmental imbalance or developmental deviation (35). Our study also showed that language delay was the most common phenomenon among children with GDD. This is similar to the results of Liao et al. (33). The adaptability DQ, gross motor DQ, fine motor DQ and personal social skills DQ scores of GDS were mainly slightly impaired, and the language ability was severe and extremely severe defected. The language ability has predictive value for the language IQ of children with GDD (34). This reminds us that in order to optimize the developmental outcomes of the children with GDD, special attention should be paid to the training programs of their language ability.

### The Association Between DQ and Autistic Symptoms in Children With GDD

There are few studies on the relationship between autism symptoms and developmental quotient in children with GDD. In our study, we found that adaptability DQ, fine motor DQ, language DQ, personal social skills DQ and total average DQ were all negatively correlated with the symptoms of autism in children with GDD. The total scores of CARS was negatively correlated with gross motor DQ, but there was no correlation between the total scores of the ABC and gross motor DQ. Our results suggested that the lower the overall developmental level, the more severe autistic symptoms in children with GDD. This is comparable to the previous findings that ID with ASD is more severe than ID alone (16). In addition, several studies have also shown that even with intensive interventions, children with ASD and comorbid cognitive delays seemed to make only limited developmental progress over time (36, 37).This again emphasizes that it is important to pay attention to the comorbidity of ASD and GDD in the early stages of development.

### Developmental Features of GDD^+^ASD^−^ Group and GDD^+^ASD^+^ Group

Regarding the development profile of children in the GDD^+^ASD^−^ and GDD^+^ASD^+^ subgroups, it was found that the developmental characteristics of the two subgroups had some common points, but there were also some differences. Language delay was the most obvious defect of the both groups, followed by personal social activity. In the GDD^+^ASD^+^ group, the gross motor DQ score was better than fine motor DQ score, fine motor DQ score was subsequently better than adaptability DQ score. There was no significant difference in gross motor, fine motor and adaptability DQ scores in the GDD^+^ASD^−^group. Few studies on the developmental features of GDD^+^ASD^−^ and GDD^+^ASD^+^ are available for comparison. In 2005, Indian scholars conducted a comparative study on 64 children under 4 years of age with ASD and developmental delays. They found significant differences in the developmental profiles of the two groups. Compared with the developmental delay group, the significantly lower social skills and superior motor ability in the ASD group explained these differences. No common features were found in the developmental profiles of the two groups (38). As the etiology and symptoms of GDD and ASD often overlap, it is sometimes difficult to distinguish between the two conditions in the clinical practice (6, 7). Although there are a variety of screening tools available and some specific clinical symptoms are helpful for the differential diagnosis of GDD and ASD, there are still certain limitations (7, 39, 40). Research by Miller et al. found that children with a developmental level of <12 months, even if the standardized ASD diagnostic tool ADOS was used for assessment, 18.8% of children with GDD were still overestimated as ASD (7). Furthermore, the inaccurate diagnosis directly affects the follow-up rehabilitation treatment. Our study showed that children in both GDD^+^ASD^−^ group and GDD^+^ASD^+^ group had similarities in their developmental profiles, but there were also clear differences, which could be helpful in the differential diagnosis of the two conditions.

Comparing development quotient of GDD^+^ASD^−^ group and GDD^+^ASD^+^ group, we found that the developmental level of children with GDD^+^ASD^+^ was more impaired than children with GDD^+^ASD^−^. The adaptability, fine motor, language and personal social skills DQ scores of GDD^+^ASD^+^ children were lower than GDD^+^ASD^−^ children, but gross motor DQ score of GDD^+^ASD^+^ children was better than GDD^+^ASD^−^ children. A study conducted by the CDC also found that children with ASD and ID had a higher proportion of developmental problems before the age of 3 compared with children with ASD alone (36). However, children with ASD and comorbid cognitive delays appeared to make limited developmental progress over time, and even under intensive intervention, they showed serious deficits in adaptive functioning, social skills, and disruptive behaviors (41, 42). These findings again indicate that special attention should be paid to the comorbidities of GDD and ASD in the early stages of development, and targeted rehabilitation should be carried out in a timely manner according to their development characteristics to improve the prognosis.

### Strengths and Limitations

This study is the first report on the comorbidity of ASD among children with GDD in China. This first report of the comorbidity rate of ASD in GDD, summarized the similarities and differences between GDD^+^ASD^−^ and GDD^+^ASD^+^ children's developmental profiles, and found that the level of development was related to the symptoms of autism in GDD. Our research also has some limitations. This is a single-center cross-sectional study, and the available results cannot represent the overall situation and dynamic changes of Chinese children with GDD. Our study only compared the developmental characteristics of children with GDD alone and GDD children with ASD, but did not include children with ASD alone. Therefore, in order to achieve the early differential diagnosis and precise intervention for children with GDD/ASD, a multi-center and longitudinal follow-up study should be conducted for the entire population of ASD and GDD.

## Conclusion

In conclusion, we found that the comorbidity rate of ASD in children with GDD was 62.3%; GDD children with poor developmental levels were more likely to have ASD symptoms. The GDD^+^ASD^−^ group and GDD^+^ASD^+^ group had not only similarities, but also differences in the developmental features; The overall development level of GDD^+^ASD^+^ children was worse than that of GDD^+^ASD^−^ children. These results will be helpful to discover more effective methods to identify ASD comorbidities in children with GDD.

## Data Availability Statement

The raw data supporting the conclusions of this article will be made available by the authors, without undue reservation.

## Ethics Statement

The studies involving human participants were reviewed and approved by the Ethics Committee of the First Hospital of Jilin University. Written informed consent to participate in this study was provided by the participants' legal guardian/next of kin.

## Author Contributions

LS, F-YJ, and Z-DX participated in the design and definition of this study. F-YJ provided assistance for data acquisition and literature search. LS and T-TW performed the statistical analysis and drafted the manuscript. LS, F-YJ, and Z-DX edited the manuscript. All authors contributed to the article and approved the submitted version.

## Funding

This work was funded by the National Natural Science Foundation of China (No: 81973054), the Key Field Research and Development Program of Guangdong Province (No: 2018B030335001), the National Key Research and Development Project of China (No: 2016YFC1306204), and the Health Special Program of the Department of Finance of Jilin Province (No: 2018SCZWSZX-060).

## Conflict of Interest

The authors declare that the research was conducted in the absence of any commercial or financial relationships that could be construed as a potential conflict of interest.

## Publisher's Note

All claims expressed in this article are solely those of the authors and do not necessarily represent those of their affiliated organizations, or those of the publisher, the editors and the reviewers. Any product that may be evaluated in this article, or claim that may be made by its manufacturer, is not guaranteed or endorsed by the publisher.
